# Effects of exogenous glucose on *Pseudomonas aeruginosa* biofilm formation and antibiotic resistance

**DOI:** 10.1002/mbo3.933

**Published:** 2019-09-18

**Authors:** Pengfei She, Yanle Wang, Yiqing Liu, Fang Tan, Lihua Chen, Zhen Luo, Yong Wu

**Affiliations:** ^1^ Department of Clinical Laboratory The Third Xiangya Hospital of Central South University Changsha China

**Keywords:** antibiotic resistance, biofilm, extracellular polysaccharide, glucose, metabolomics, *Pseudomonas aeruginosa*

## Abstract

*Pseudomonas aeruginosa* is commonly found in nosocomial and life‐threatening infections in patients. Biofilms formed by *P. aeruginosa* exhibit much greater resistance to antibiotics than the planktonic form of the bacteria. Few groups have studied the effects of glucose, a major carbon source, and metabolite, on *P. aeruginosa* biofilm formation and on its metabolic pathways. In this study, we investigated the effect of glucose on the biofilm formation ability of *P. aeruginosa* and carried out a metabolomic analysis to identify whether glucose alters the metabolic activity of *P. aeruginosa* in biofilms. We found that glucose efficiently promoted *P. aeruginosa* biofilm formation by upregulating the expression of the extracellular polysaccharide‐related gene *pslA*. Treatment with glucose caused an increase in 7 metabolites (including 3‐hydroxypropionic acid, glucose‐6‐phosphate, and 2,3‐dimethylsuccinic acid) and a decrease in 18 metabolites (including myo‐inositol, glutamine, and methoxamedrine) in the biofilm. In addition, there was a synergistic effect between glucose and horse serum on biofilm formation when the two were added in combination, which also increased the resistance of biofilm to levofloxacin therapy. Thus, our work sheds light on the underlying mechanisms by which glucose may enhance biofilm formation and identifies novel targets for developing strategies to counteract biofilm formation.

## Graphical abstract

Glucose causes metabolic changes associated with biofilm formation. Higher dose of levofloxacin is needed to eliminate biofilms formed in the presence of glucose.

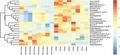

## 1 INTRODUCTION

Bacterial species can grow in a single‐celled planktonic form or in a physiologically distinct biofilm form. A biofilm is composed of layers of bacteria within a hydrated matrix consisting of extracellular polysaccharide (EPS), extracellular DNA, proteins, and lipids (Costerton et al., 1987; Laverty, Gorman, & Gilmore, 2013). Gene expression and metabolic activity varies significantly between biofilm and the planktonic bacterial growth forms (Stoodley, Sauer, Davies, & Costerton, 2002). Biofilms are notoriously difficult to eradicate and are responsible for many recalcitrant infections with important clinical consequences (Donlan, 2002). The National Institute of Health of the USA currently estimates that biofilms account for over 80% of all infections in the human body (Harriott & Noverr, 2009).

*Pseudomonas aeruginosa* is a gram‐negative opportunistic pathogen commonly found in nosocomial and life‐threatening infections in patients with cystic fibrosis (CF) (Van Delden & Iglewski, 1998) and demonstrates extensive antibiotic resistance. In immunocompromised individuals, *P. aeruginosa* proliferates in a self‐produced glycocalyx and forms a biofilm that imparts antibiotic resistance (Wei & Ma, 2013). *Pseudomonas aeruginosa* biofilm formation includes four major steps: surface attachment, microcolony formation, maturation, and dispersion (Whiteley et al., 2001). Published reports suggest that *P. aeruginosa* forms biofilms that confer a survival advantage to bacteria, such as increased antibiotic tolerance in the lungs of CF patients (Mah & O'Toole, 2001).

The extracellular matrix of *P. aeruginosa* is responsible for biofilm architecture and functions as an adhesive, holding biofilm cells together and protecting them against antibiotics, metallic cations, ultraviolet radiation, oxidizing biocides, and host immune defenses (Flemming & Wingender, 2010; Stoodley et al., 2002). Extracellular polysaccharide is a major component of the *P. aeruginosa* biofilm extracellular matrix (Flemming & Wingender, 2010). *P. aeruginosa* produces at least three different types of EPS: Pel, Psl, and alginate which contribute to biofilm formation and architecture maintenance (Ryder, Byrd, & Wozniak, 2007). Psl consists of a repeated pentasaccharide containing D‐mannose, D‐glucose, and D‐rhamnose and plays a vital role in the adherence to abiotic and biotic surfaces and in the maintenance of biofilm architecture. Pel, a glucose‐rich polysaccharide, is important for biofilm formation in air–liquid interfaces (Byrd et al., 2009). In contrast, alginate exists only in the mucoid *P. aeruginosa* and is essential for the initial steps of biofilm development (Byrd et al., 2009). Although glucose and its derivatives are the major components of EPS, only few reports have demonstrated the effects and mechanisms of exogenous glucose on *P. aeruginosa* biofilm formation. Understanding the molecular mechanism of glucose‐induced biofilm formation may help in identifying novel targets to eradicate *P. aeruginosa* biofilms in CF patients or in environmental conditions associated with high levels of glucose and biofilms.

In recent years, many factors such as ethanol, glucosamine, temperature, and subinhibitory concentrations of certain antibiotics have been reported to influence extracellular matrix expression and biofilm formation in vitro (You et al., 2014). As a carbon source and a metabolite, glucose shows multiple effects on bacterial growth and biofilm formation. You et al. (2014) found that glucose induced *Staphylococcus aureus* biofilm formation through the accessory protein GbaAB in a polysaccharide intercellular adhesion‐dependent manner. Pan, Breidt, and Gorski (2010) reported that glucose combined with sodium chloride showed a synergistic effect on promoting *Listeria monocytogenes* biofilm formation through the accumulation of extracellular polymeric substances rather than by increasing the number of viable biofilm cells. However, the effects of glucose on *P. aeruginosa* biofilm formation and associated metabolic pathway alterations, as assessed by metabolomics, have not been analyzed.

In the present study, we investigated the effects of glucose on biofilm formation by *P. aeruginosa* strain PAO1 and clinical isolates using a multiphenotypic approach and metabolomics.

## 2 MATERIALS AND METHODS

### 2.1 Bacterial strains and plasmids

The PAO1 strain (ATCC 15692) used in this study was kindly provided by Mingqiang Qiao (College of Life Sciences of Nankai University, Tianjin, P.R. China). *P. aeruginosa* PA47 is a clinical isolate and strong biofilm producer, as we previously reported (She et al., 2018). Other clinical isolates of *P. aeruginosa* were collected from patients with pulmonary infections at the Third Xiangya Hospital of Central South University (Changsha, Hunan, P.R. China) from January 2014 to December 2014 (Qu et al., 2016). Detailed information on other strains and plasmids used in this study is shown in Table 1. All strains were stored at −80°C in whole milk culture. Glucose‐free Luria‐Bertani (LB) broth (Solarbio) was used for bacterial planktonic cell growth and biofilm formation in all experiments; Mueller‐Hinton (MH) broth (Solarbio) was used for the antibiotic susceptibility assay.

**Table 1:** Strains and plasmids used in this study

Name	Genotype or description	Source
Strain		
ATCC15692 (PAO1)	Sequenced strain	Xu et al. (2005)
PA47	Clinical strain; Strong biofilm formation ability	Qu et al. (2016)
PA47*∆pslA*	Unmarked deletion of *pslA*	This laboratory
PA01	Clinical strain	Qu et al. (2016)
PA07	Clinical strain	Qu et al. (2016)
PA09	Clinical strain	Qu et al. (2016)
*E. coli* DH5α	*E. coli* strain; F^‐^φ80*lacZ ∆M15 recA1 and A1 hsdR17 supE44 thi−1 gyrA96 relA1 (lacZYA‐argF) U169 λpir lysogen*	Luo, He, Dou, Zhang, and Shen (2015)
Plasmid		
pLP12	Vector; vmi480; chloramphenicol^R^(Chl^R^)	Demarre et al. (2005)
pLP12‐∆pslA	pLP derivative for unmarked deletion of pslA; Chl^R^	This laboratory

### 2.2 *P. aeruginosa* PA47∆pslA construction

A 573‐bp DNA fragment containing the upstream region of *pslA* was amplified using primers PslA‐MF1 and PslA‐MR1. A 556‐bp DNA fragment containing the *pslA* downstream region was amplified using primers PslA‐MF2 and PslA‐MR2. The two PCR products were then amplified using PrimerSTAR Max DNA Polymerase (TaKaRa). A 1,129‐bp DNA fragment of the PCR product was ligated into pLP12 with Exnase II (ClonExpress II, Vazyme) to generate pLP12‐*∆pslA* and was further confirmed by primers pLP‐UF and pLP‐UR. DH5α and PA47 wild‐type strains were transformed with this plasmid. Deletion of *pslA* was confirmed by PCR using primers PslA‐MF1 and PslA‐MR2. Detailed information about the strains and primers is shown in Table 1 and Table A1, respectively.

### 2.3 Biofilm determination

An overnight culture of *P. aeruginosa* was diluted 200‐fold with LB containing twofold serially diluted glucose or levofloxacin (OFLX) or horse serum (HoS), and 200 μl of the suspension was added into microplates. After incubation at 37°C for 24 hr without shaking, the planktonic cells were removed by saline washing, and the biofilm cells adhering to the wells were stained by crystal violet or XTT [2,3‐bis‐(2‐methoxy‐4‐nitro‐5‐sulfophenyl)‐2H‐tetrazolium‐5‐carboxanilide].

For crystal violet staining (She et al., 2018), 200 µl of 0.25% (wt/vol) crystal violet was added to each well and incubated at room temperature for 15 min. Unbound dye was removed by saline washing. The plates were allowed to air dry, and 95% ethanol was added to dissolve the bound dye. After incubation at room temperature for 20 min, the absorbance of the ethanol at 570 nm (A570) was measured by a microplate spectrophotometer (Bio‐Rad, USA).

For XTT staining assay (Psoter, Rosenfeld, De Roos, Mayer, & Wakefield, 2014)**,** XTT was diluted with 1 × PBS (pH = 7.0) to a final concentration of 0.2 mg/ml and mixed with phenazine methosulfate (0.02 mg/ml). Then, 200 μl of this mixture was added to each well, and after incubation at 37°C for 3 hr in the dark, the absorbance at 490 nm (A490) was measured.

### 2.4 Growth curve

Glucose was diluted with LB broth to a final concentration of 1%–4%. Overnight cultured PA47 was added to the glucose at a final concentration of ~5 × 10^5^ CFU/ml. After incubation at 37°C (180 rpm), 200 μl of bacterial suspension was added to microplates at 0, 8, 16, and 24 hr, and the absorbance at 630 nm (A630) was measured (She et al., 2018).

### 2.5 Confocal laser scanning microscope (CLSM) imaging and analysis

Biofilms of PA47 were grown on coverslips in the presence or absence of 2% or 4% glucose as described above. Three fluorescent dyes (SYTO9 for total biomass, propidium iodide for dead cells, and ConA for α‐polysaccharides; Thermo Fisher Scientific) were added to each coverslip, and after staining in the dark, samples were imaged using a CLSM (Zeiss LSM 800). The excitation and emission wavelengths for the three dyes were as follows: SYTO9: 633 nm and 650–700 nm; propidium iodide: 458 nm and 460–500 nm; and ConA: 543 nm and 550–600 nm; ImageJ software was used to quantify the biofilm biomass (Qu et al., 2016).

### 2.6 Extracellular polysaccharide analysis by phenol‐sulfuric acid

Biofilms of PA47 with or without 4% glucose treatment were constructed in 6‐well cell culture plates. The planktonic cells and free glucose were thoroughly removed by washing five times with saline and the biofilms were scraped into 0.5 ml saline with moist swabs. After mixing, 0.5 ml of phenol (5%) and 5 ml of concentrated sulfuric acid was added and incubated for 1 hr in the dark, and the A490 was measured (Musthafa, Sivamaruthi, Pandian, & Ravi, 2012).

### 2.7 qRT‐PCR

Gene expression was detected by qRT‐PCR according to our previous study (Qu et al., 2016). Briefly, overnight cultures of PA47 grown in LB broth in the presence or absence of 2% glucose were collected at the A630 of 0.5–0.8. Total RNA was extracted using an E.Z.N.A. Total RNA Kit II (Omega Bio‐tek). The RNA purity and concentration was determined by the absorbance at 260/280 nm, and 1 μl of RNA was used for cDNA synthesis by TransScript All‐in‐One First‐Strand cDNA Synthesis SuperMix (Transgene). qPCR was performed using TransStartTM Green qPCR SuperMix UDG (Transgene) using a real‐time quantitative PCR system (Eppendorf). The oligonucleotide primers used to amplify the housekeeping gene 16S rRNA and EPS‐related genes (*pelA*, *pslA,* and *alg44*) (Kim, Park, & Lee, 2015) are shown in Table A1.

### 2.8 Metabolomics analysis

#### 2.8.1 Metabolite extraction

Overnight culture of 8 *P. aeruginosa* clinical isolates was diluted with LB broth to ~10^6^ CFU/ml in the presence/absence of 4% glucose in a 6‐well microplate. After incubation at 37°C for 24 hr, the planktonic cells were removed; the remaining biofilms were collected with a cell scraper and washed four times with 1 × PBS, and then subjected to metabolomics analysis. Briefly, samples were collected in 2‐ml tubes and extracted with 1 ml of extraction reagent (V_Methanol_: V_Chloroform_ = 3:1). After vortexing for 30 s, samples were homogenized using a ball mill for 4 min at 45 Hz and then treated with ultrasound 5 times for 5 min each (incubated in ice water). After centrifugation for 15 min at ~12,000  *g*, and 4°C, the supernatant (0.9 ml) was transferred to a 2 ml GC/MS glass vial. After the sample was completely dried in a vacuum concentrator with no heating, 60 μl of methoxyamine hydrochloride (20 mg/ml in pyridine) was added to the sample and incubated for 30 min at 80°C. Next, 80 μl of BSTFA reagent (1% TMCS, vol/vol) was added to the samples and incubated for 1.5 hr at 70°C. All samples were analyzed by a gas chromatograph system coupled with a Pegasus HT time‐of‐flight mass spectrometer (GC‐TOF‐MS).

#### 2.8.2 GC‐TOF‐MS analysis

GC‐TOF‐MS analysis was performed using an Agilent 7890 gas chromatograph system coupled with a Pegasus HT time‐of‐flight mass spectrometer. The system utilized a DB‐5MS capillary column coated with 5% diphenyl cross‐linked with 95% dimethylpolysiloxane (30 m × 250 μm inner diameter, 0.25 μm film thickness; J&W Scientific). An 1 μl aliquot of the analyte was injected in splitless mode. Helium was used as a carrier gas; the front inlet purge flow was 3 ml per min, and the gas flow rate through the column was 1 ml per min. The initial temperature was kept at 50°C for 1 min, then raised to 310°C at a rate of 10°C per min, and then maintained at 310°C for 8 min. The injection, transfer line, and ion source temperatures were 280, 280, and 250°C, respectively. The energy was −70 eV in electron impact mode. Mass spectrometry data were acquired in full‐scan mode with the m/z range of 50–500 at a rate of 20 spectra per second after a solvent delay of 6.27 min (Garcia & Barbas, 2011; Kind et al., 2009).

### 2.9 Minimal inhibitory concentration (MIC) and minimal biofilm eradication concentration (MBEC)

#### 2.9.1 MIC determination

The MIC was detected by the broth microdilution method as previously described by the Clinical and Laboratory Standards Institute 2015 (CLSI, 2015). Briefly, twofold dilutions of OFLX (ranging from 1,024 to 0.0625 μg/ml) were made in 96‐well plates with 100 μl of MH or LB broth per well. A bacterial suspension (0.5 McFarland 1/20 diluted, 10 μL) was added, and plates were incubated at 37°C for 16–18 hr. The MIC was determined as the lowest concentration without any visible bacterial growth.

#### 2.9.2 MBEC determination

Minimal biofilm eradication concentration was assessed by the XTT staining method as described above. Briefly, overnight cultures were added to microplates with or without 4% glucose at 37°C for 24 hr. After removing the planktonic cells with saline, LB broth containing different concentrations of OFLX (ranging from 1,024 to 0.0625 μg/ml) were added to each well. After incubation for 24 hr, planktonic cells were removed by saline washing, and XTT combined with phenazine methosulfate was added to each well as described above. The MBEC_50_ and MBEC_90_ were defined as the eradication of 50% and 90% of the 24 hr preformed biofilm, respectively, when compared to the untreated controls.

### 2.10 Statistical analysis

Data were analyzed by GraphPad Prism 7.0 software. For all figures with bar graphs, the data are represented as the mean ± standard deviation. ANOVA and Student's *t* test were used to determine the statistical significance. A *p*‐value <.05 was considered indicative of statistical significance. For metabolomics data preprocessing and annotation, Chroma TOF 4.3X software (LECO Corporation) and the LECO‐Fiehn Rtx5 database were used for raw peak extraction, baseline data filtering, and calibration of the baseline, peak alignment, deconvolution analysis, peak identification, and integration of the peak area. Both the retention index and mass spectrum match were considered in metabolite identification.

## 3 RESULTS

### 3.1 Biofilms of *P. aeruginosa* clinical isolates are enhanced by glucose treatment

**Figure 1:**
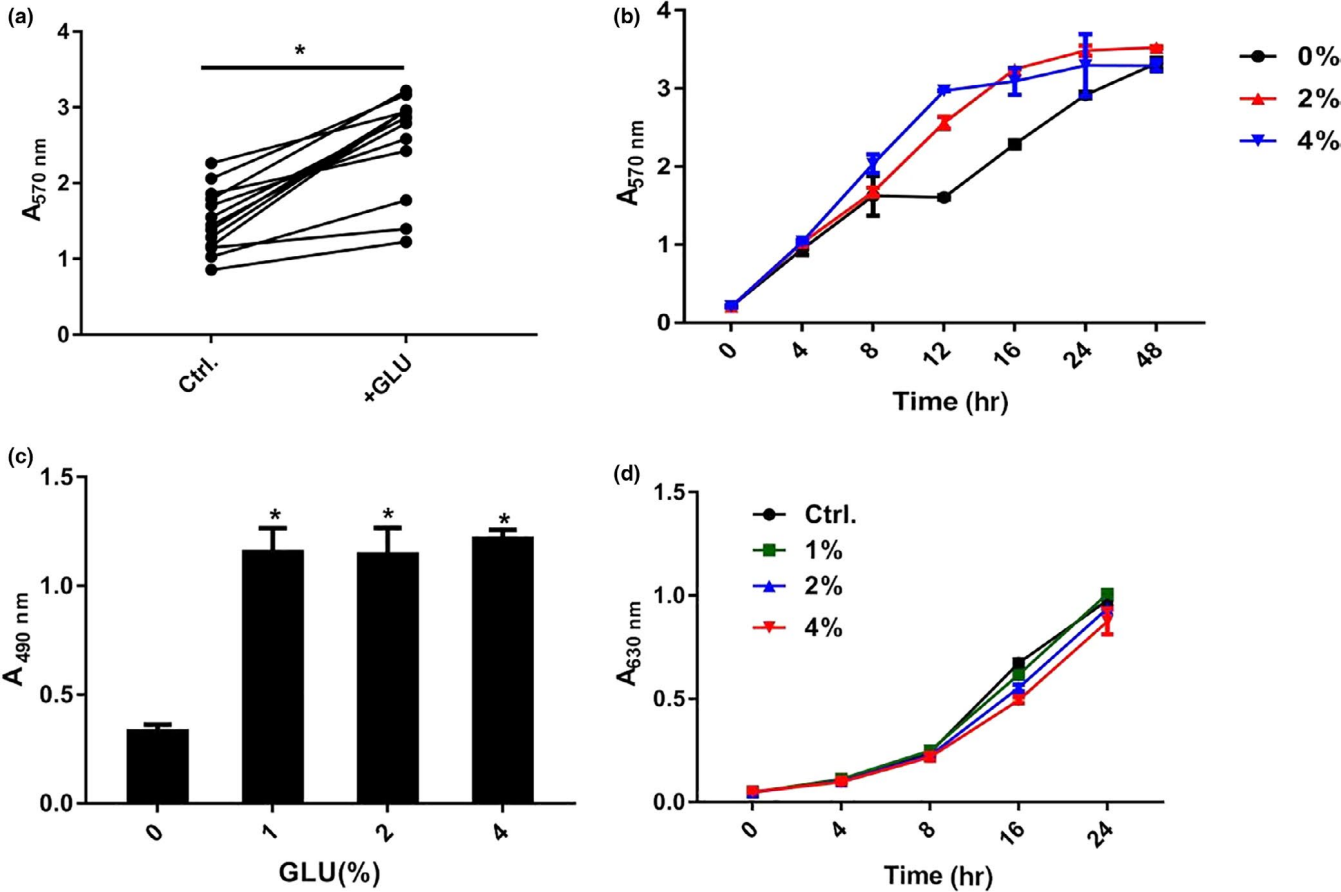
Effects of glucose on biofilm formation by *P. aeruginosa* clinical isolates. (a) Effects of 2% glucose on the biofilm formation of *P. aeruginosa* clinical isolates. Each plot represents the average of 3 experimental replicates. (b) Crystal violet staining of the PA47 biofilm in the presence of 2% and 4% glucose at various time points. (c) Effect of glucose on PA47 biofilm metabolic activity by XTT assay. (d) Growth curve of PA47 in the presence or absence of glucose at concentrations ranging from 1% to 4%. *p < .05, compared with the untreated control group. These experiments were independently repeated three times in triplicate

The biofilm biomass of *P. aeruginosa* was determined by crystal violet staining and XTT assay. Though the clinical isolates showed different sensitivities to glucose treatment, we found that glucose increased the biofilm formation in all of the tested *P. aeruginosa* strains (PAO1 and 12 clinical isolates) at a concentration of 2% (Figure 1a). The isolate PA47 was selected for subsequent experiments based on its response to glucose. As depicted in Figures 1b, 2% and 4% glucose significantly enhanced biofilm formation by PA47 in a time‐dependent manner from 8–24 hr treatment (*p* < .05) when compared to no glucose addition. However, biofilm growth plateaued after 48 hr incubation (Figure 1b). Although total biofilm biomass was assessed by crystal violet staining, the XTT staining assay determined the metabolic activity of live cells in biofilms. We also found that glucose significantly enhanced the metabolic activity of biofilms at a concentration of 1%–4% (Figure 1c). Interestingly, planktonic cell growth was not influenced by up to 4% glucose (Figure 1d), indicating that glucose enhances biofilm formation by upregulating the extracellular matrix and enhancing cell attachment rather than increasing bacterial proliferation. A previous study by Yang et al. (2018) reported that different attachment phenotypes of planktonic cells mainly result from the distinct production of the polysaccharide Psl.

**Figure 2:**
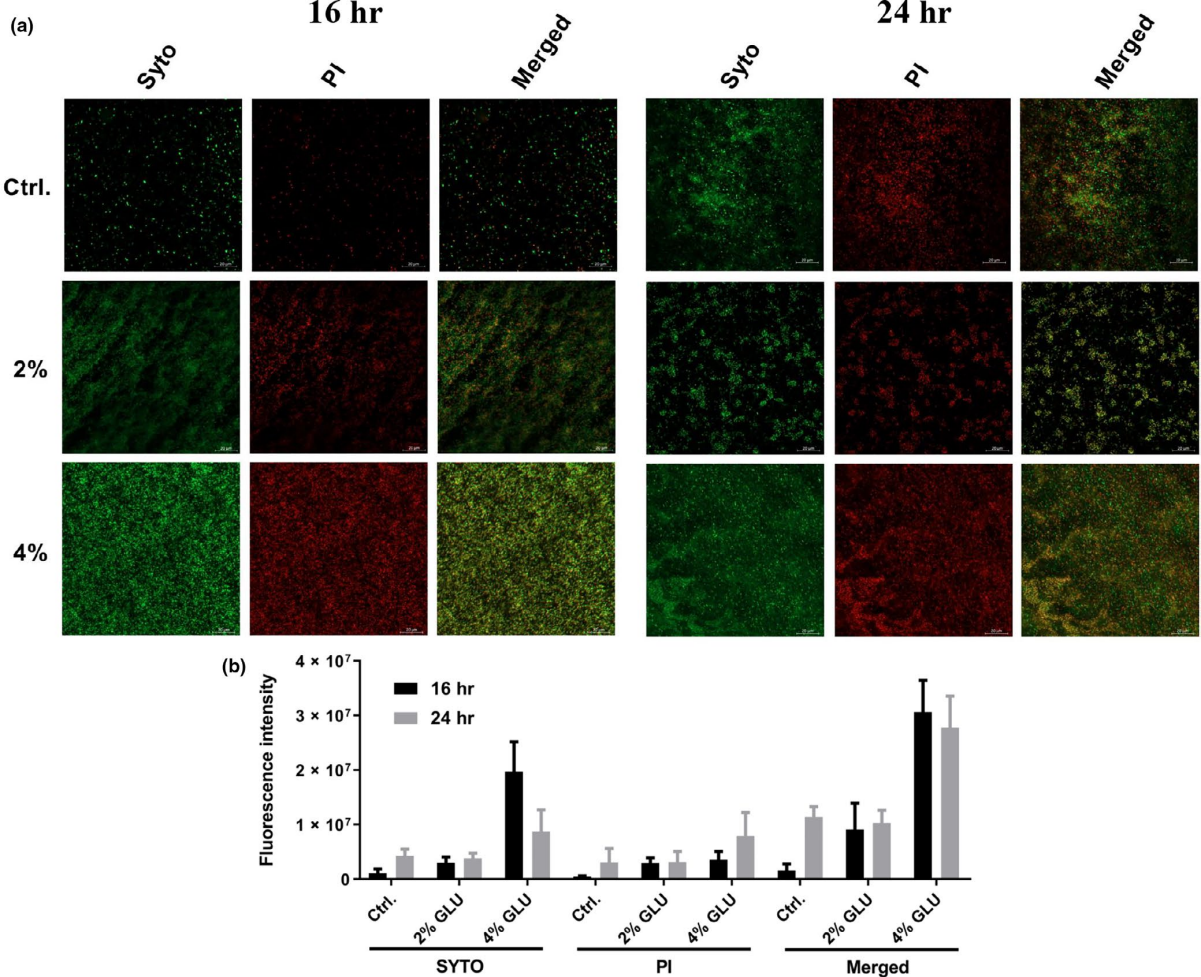
CLSM imaging of PA47. (a) CLSM observation by LIVE/DEAD BacLight Bacterial Viability Kit. The live cells stained with SYTO9 (green), and the dead cells are stained with propidium iodide (red). Scale bar, 20 μm. (b) ImageJ analysis of the coverage ratios of total/live/dead cells in biofilm biomass. The experiment was independently repeated three times in triplicate

Confocal laser scanning microscope imaging showed live (green) and dead/membrane damaged (red) cells as well as morphological biofilm changes in the presence or absence of 2% and 4% glucose (Figure 2a). In accordance with the above observations, 2% glucose significantly enhanced biofilm formation of the PA47 strain at 16 hr, while no biofilm biomass enhancement effect was observed after 24 hr of incubation in the presence of 2% glucose (Figure 2b). In contrast, 4% glucose significantly increased biofilm formation of PA47 both at 16 hr and 24 hr (Figure 2a,b). Noticeably, the fluorescence intensity of PI was slightly increased in Figure 2b for both 16 hr and 24 hr glucose treatment. This probably cannot be only duo eo the increasing number of dead cells since the live cells with increased membrane permeability could also be stained by PI. And in the process of biofilm maturation, the inner cells in the biofilms could be transferred to the state of low metabolic activity and even dead cells, and the components of dead cells could be reused to build biofilms.

### 3.2 Glucose increases EPS production by upregulating *pslA* gene expression

**Figure 3:**
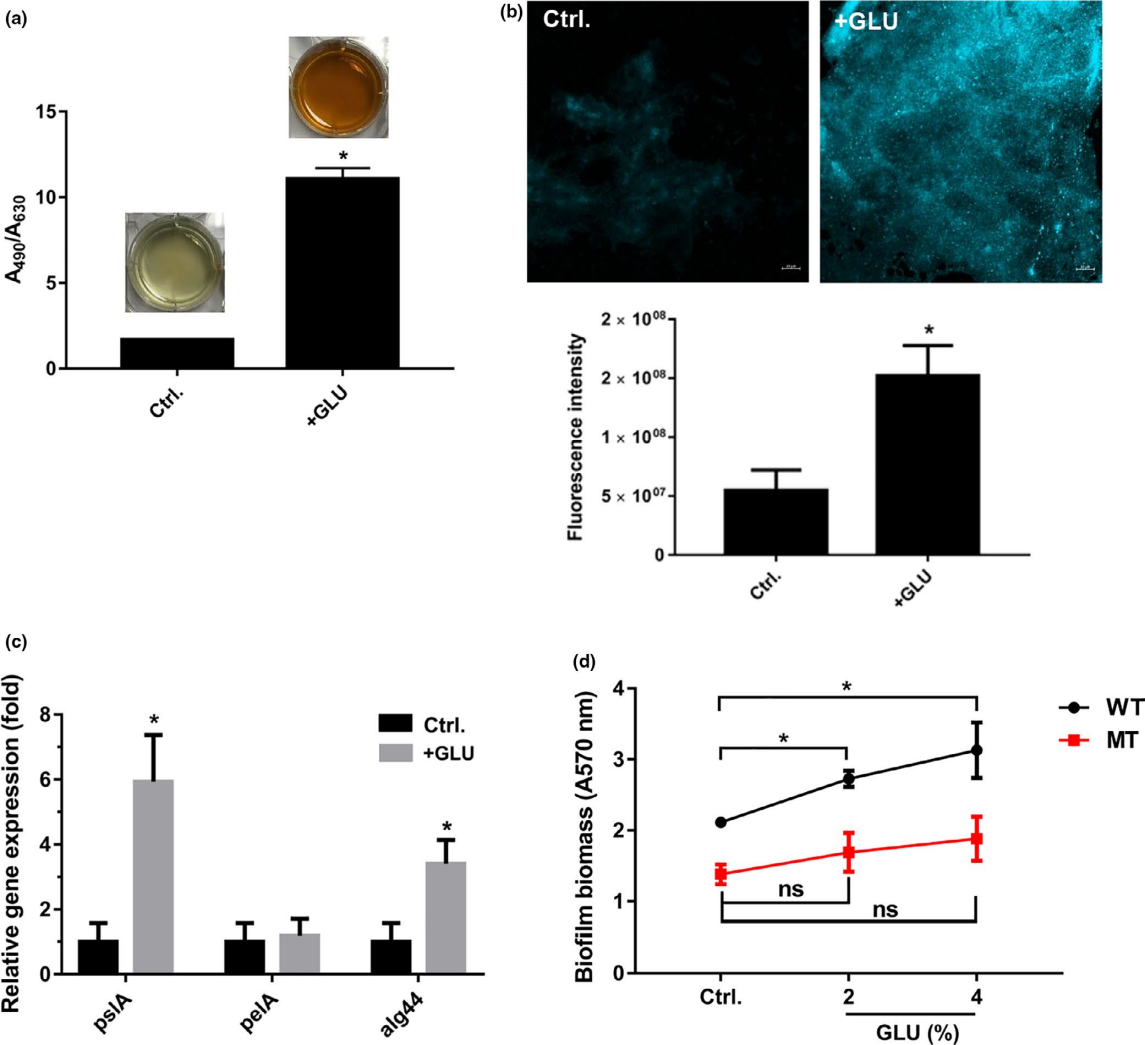
Effects of glucose on the production of EPS by PA47. (a) EPS production was determined by phenol‐sulfuric acid assay. (b) CLSM observation and coverage ratio analysis of EPS by ConA staining. (c) glucose at 4% final increased the expression of the EPS‐related genes *pslA* and *alg44* by qRT‐PCR analysis. The housekeeping gene 16S rRNA was used for normalization. (d) Effect of glucose on biofilm formation in PA47 or PA47⊿*pslA*. ns, no statistical significance; *p < .05, compared with the untreated control group. These experiments were independently repeated three times in triplicate

Extracellular polysaccharide is a major component of the biofilm extracellular matrix. Increased secretion of the polysaccharide was confirmed by a phenol/concentrated sulfuric acid assay in 6‐well microplates (Figure 3a). Moreover, since the fluorescent dye ConA binds to the α‐mannopyranosyl and α‐glucopyranosyl sugars in the biofilm matrix (Chen, Lee, Tay, & Show, 2007), the CLSM images and ImageJ analysis showed that the EPS was markedly increased in the presence of 4% glucose (Figure 3b). Using the housekeeping gene 16S rRNA as reference, qRT‐PCR analysis showed that compared with the no glucose control, 4% glucose significantly increased the expression of *pslA* (PslA) and *alg44* (alginate) genes by 6.0‐fold (*p* < .05) and 3.2‐fold (*p* < .05), respectively, without influencing *pelA* (PelA) gene expression (*p* > .05) (Figure 3c). Based on these findings, we constructed a *pslA* knockout strain of PA47⊿*pslA*. We found that compared to that of the untreated group, the biofilm biomass was significantly increased in the wild‐type PA47 group in the presence of 2% and 4% glucose (*p* < .05) at 16 hr, but there was no significant difference in the PA47⊿*pslA* group in the presence of glucose (Figure 3d). So, we assumed that glucose could increase the biofilm formation of *P. aeruginosa* by enhancing *pslA* gene expression in a moderate dosing‐dependent manner.

### 3.3 Glucose alters the metabolic pathways in *P. aeruginosa*

**Figure 4:**
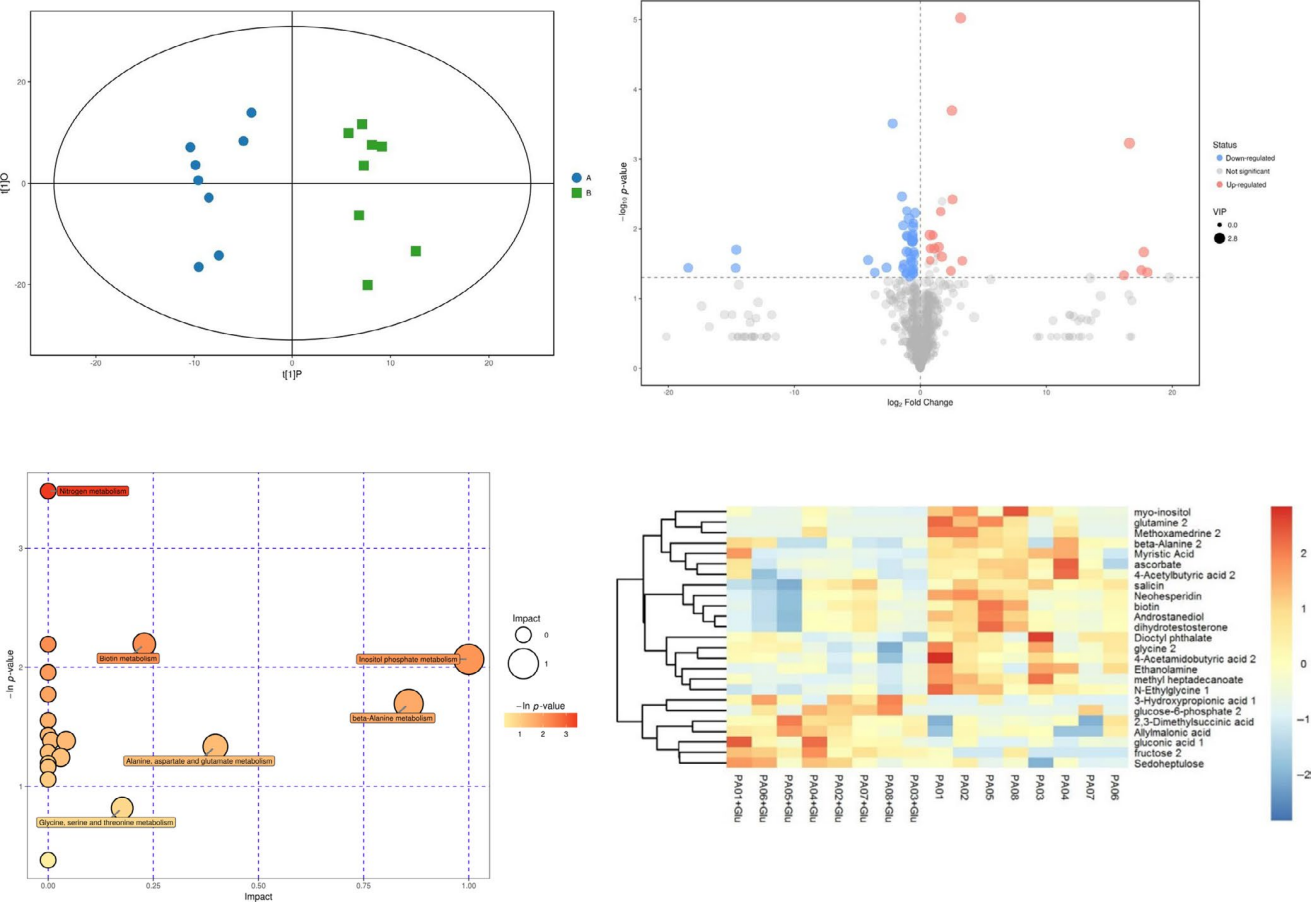
Metabolomics analysis of the *P. aeruginosa* biofilm treated with glucose. (a) Two‐dimensional principal component analysis (PCA) of 16 samples based on global metabolomic profiles. A: control group, B: glucose‐treated group. (b) Volcano plots of significantly up‐ or downregulated metabolites in response to glucose treatment. (c) GO analysis showing altered metabolic pathways in the presence of glucose. (d) Heat map and clustering showing metabolomic profiles of 16 clinical isolates of *P. aeruginosa* in the presence or absence of glucose based on 25 significantly different metabolites

By metabolomics analysis, we found that *P. aeruginosa* metabolism was significantly affected by 4% glucose treatment (Figure 4). Principal component analysis (PCA) was carried out to assess the quality of samples. Eight clinical isolates of *P. aeruginosa* in the presence or absence of glucose were divided into two groups based on their distinctive profiles in the PCA plots (Figure 4a). By using one‐way ANOVA, a total of 55 molecular features were identified to be significantly different (*p* < .05) between the two groups (Table A2). Among these features, 18 were upregulated and 37 were downregulated (Figure 4b). These metabolites (in relative intracellular metabolite concentrations of compounds) are predominantly involved in the inositol phosphate, beta‐alanine, alanine, aspartate, glutamate, glycine, serine, threonine, and biotin metabolism. As depicted in the bubble in Figure 4c, the degree of glucose impact on the metabolic pathways was positively correlated with the size of the circles and the *p* value was positively correlated with the color depth. Among the 55 features that were altered by glucose treatment, 25 metabolites were in the Fiehn database based on the parameters of accurate mass, isotope ratios, abundance, and spacing. The remaining 30 compounds were classified as unknown but were reproducibly detected. Despite some variations among individual samples, seven of the 25 metabolites were significantly increased (including 3‐hydroxypropionic acid, glucose‐6‐phosphate, 2,3‐dimethylsuccinic acid, allylmalonic acid, gluconic acid, fructose and sedoheptulose), and 18 were significantly decreased (including myo‐inositol, glutamine, methoxamedrine, beta‐alanine, myristic acid, ascorbate, and 4‐acetylbutyric acid) in the presence of glucose when compared with the untreated controls (Figure 4d).

### 3.4 Synergistic effect of glucose and HoS on increased resistance of *P. aeruginosa* biofilm to OFLX

**Figure 5:**
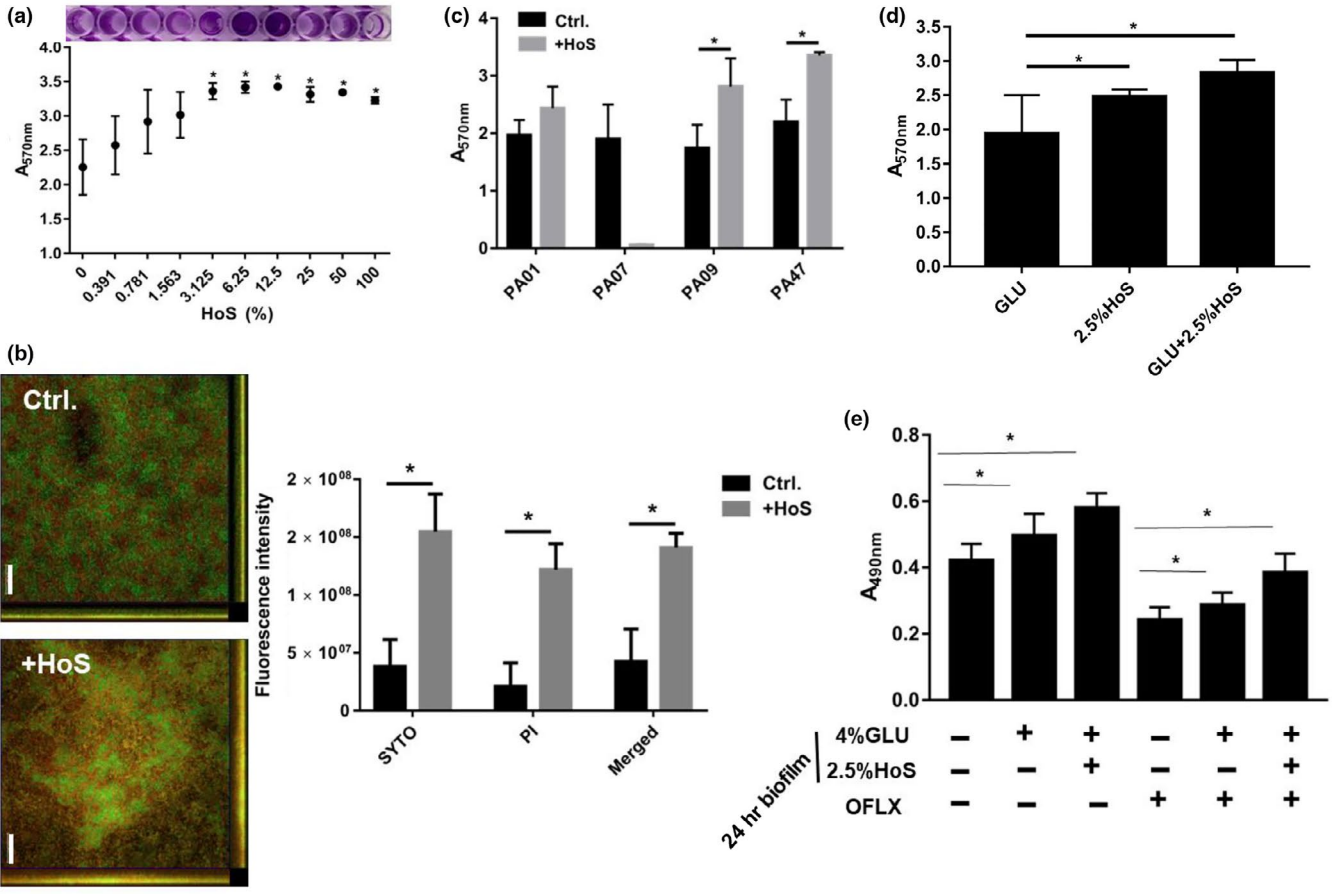
Effects of glucose on biofilm formation and resistance in the presence of HoS. (a) Effect of HoS on *P. aeruginosa* biofilm formation by crystal violet staining. (b) CLSM observation and coverage ratio analysis of glucose‐treated biofilms. Scale bar, 20 μm. (c) Biofilm formation of different clinical isolates in response to HoS treatment as assessed by crystal violet staining. (d) Synergistic biofilm‐promoting effect of HoS plus glucose by crystal violet staining. (e) Synergistic effect of glucose plus HoS on biofilm formation and OFLX resistance of *P. aeruginosa* determined by XTT staining. These experiments were independently repeated three times in triplicate

Many infections occur in the presence of serum. By crystal violet staining, we found that the presence of 3.125% HoS significantly increased *P. aeruginosa* biofilm formation (Figure 5a). CLSM imaging and ImageJ analysis revealed that HoS increased both the coverage and thickness of the biofilm biomass (Figure 5b). However, clinical isolates showed a different response to 2.5% HoS. While there was a significant inhibitory effect of 2.5% HoS on biofilm formation in PA07, HoS increased the biofilm formation by PA09 and PA47 and did not influence PA01 (Figure 5c). Interestingly, there was a significant synergistic biofilm‐promoting effect of HoS and glucose when added together (Figure 5d). By XTT staining assay, we found that the combined glucose and HoS treatment caused a significant increase in resistance to OFLX when compared to glucose treatment alone (*p* < .05) (Figure 5e).

**Figure 6:**
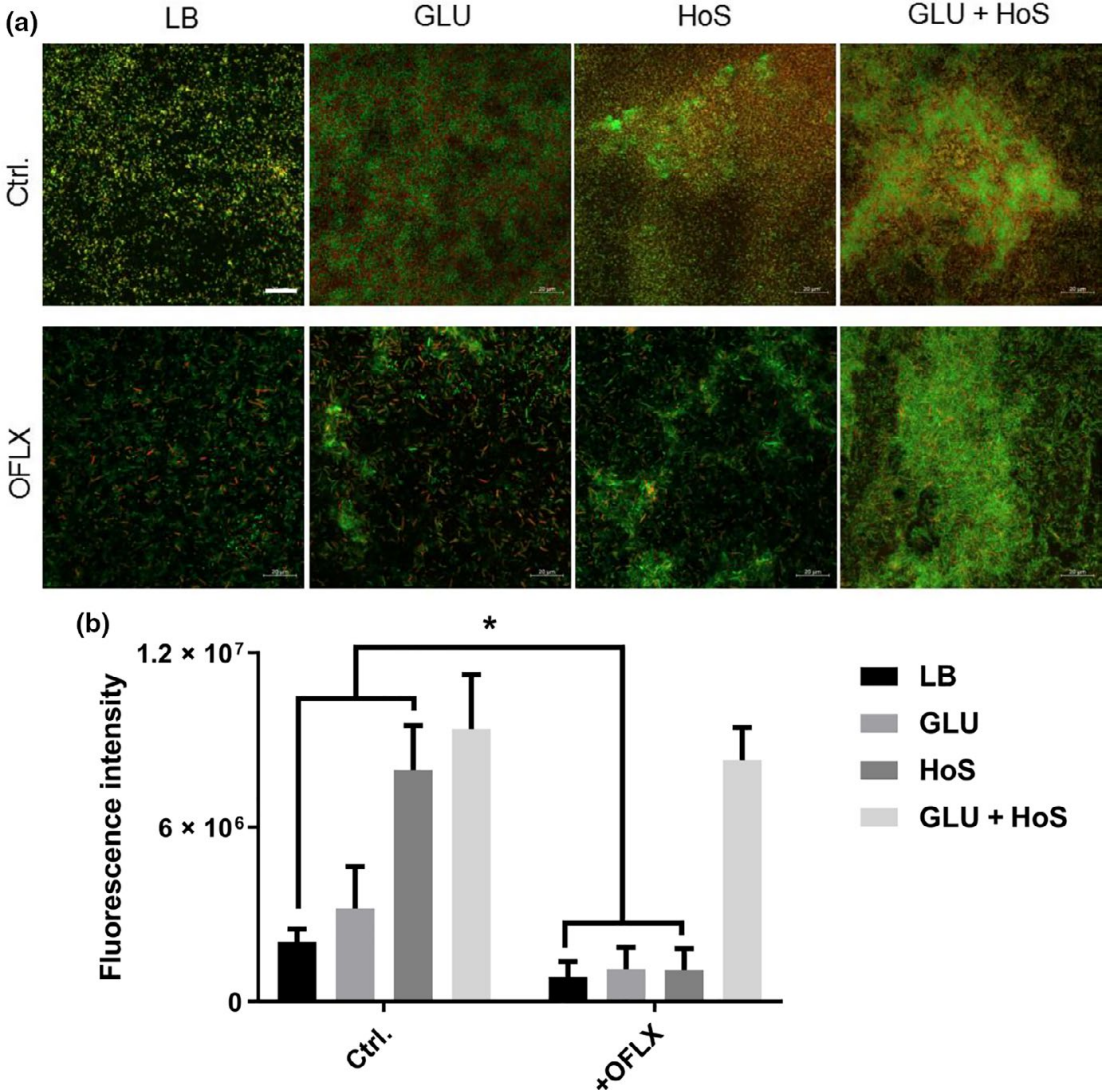
HoS and glucose increase biofilm formation and OFLX tolerance in *P. aeruginosa* PA47. (a) Biofilms in the presence/absence of glucose/HoS were treated with OFLX and stained with SYTO9 and propidium iodide was observed by CLSM. Scale bar, 20 μm. (b) The coverage ratio of the total biofilm biomass of three independent experiments was analyzed by ImageJ. *p < .05, compared with the untreated control group

This finding was further confirmed by CLSM and ImageJ analysis. Additionally, resistance to OFLX increased as biofilm formation increased in PA47, and at the same time, the percentage of live cells in the biofilm biomass also increased (Figure 6a,b). While there was a minimal effect of 2% glucose on the MICs of the clinical isolates (PA01, PA07, PA09 and PA47) in the planktonic form, glucose increased the MBEC_50_ and MBEC_90_ of these clinical isolates ranging from 2‐ to >256‐fold in the biofilm forms (Table 2).

**Table 2:** MIC and MBEC (μg/ml) of OFLX against *P. aeruginosa* in the presence/absence of 2% glucose

Strains	MIC_LB_		MIC_MH_		MBEC_50_		MBEC_90_	
	Ctrl.	+ glucose	Ctrl.	+ glucose	Ctrl.	+ glucose	Ctrl.	+ glucose
PA01	16	8	8	8	4	8	32	265
PA07	4	4	1	1	0.25	8	0.5	16
PA09	0.25	0.25	0.25	0.25	0.125	1	0.25	128
PA47	1	1	0.5	0.5	2	2	4	>1,024

Abbreviation: MBEC, minimal biofilm eradication concentration.

## 4 DISCUSSION

The present study demonstrates that exogenous glucose exhibits strong biofilm promotion and OFLX resistance activity in *P. aeruginosa* airway clinical isolates. In addition, this study provides some indications of the underlying mechanisms by which glucose promotes biofilm formation.

Extracellular polysaccharide is a major component of the extracellular polymeric matrix. The majority of EPSs include Psl, Pel, and alginate (Ryder et al., 2007). Of the many virulence factors produced by *P. aeruginosa* that exacerbate disease, alginate, Psl, and Pel are most relevant to immune evasion and chronic biofilm infections (Santos, Watanabe, & Andrade, 2011; Shanks, Sargent, Martinez, Graber, & O'Toole, 2006).

Psl is a neutral branched pentasaccharide containing d‐glucose, d‐rhamnose, and d‐mannose at a ratio of 3:1:1. Pel is a positively charged polysaccharide composed of partially acetylated 1 → 4 glycosidic linkages of *N*‐acetylgalactosamine and *N*‐acetylglucosamine (He et al., 2016). Alginate is a negatively charged hygroscopic acetylated polymer with nonrepetitive monomers of 1,4‐linked l‐guluronic and d‐mannuronic acids (Lopez, de Leon, & Moujir, 2011). The biosynthetic genes are encoded by an operon that begins with *algD* (Hoffman et al., 2005). By qRT‐PCR analysis, we found that glucose significantly increased *pslA* gene expression without influencing on *pelA* and *alg44* gene, which indicates that *P. aeruginosa* may synthesize the d‐glucose component of Psl directly or indirectly by using exogenous glucose.

Recently, evidence about the signaling role of matrix EPS has begun to emerge. Irie et al. (2012) found that the major matrix component of *P. aeruginosa*, Psl polysaccharide, serves as a signal for the production of cyclic‐di‐GMP (c‐di‐GMP), a key second messenger that drives a switch toward the biofilm phenotype in *P. aeruginosa* (Ha & O'Toole, 2015). Intracellular levels of c‐di‐GMP may be induced by supplementing cell cultures with purified Psl and by increasing *psl* expression using an inducible promoter (Irie et al., 2012).

C‐di‐GMP, like cAMP, c‐di‐AMP, cGMP, and (p)ppGpp, is an important intracellular signaling molecule (Bharati & Chatterji, 2013; Kalia et al., 2013). C‐di‐GMP is a ubiquitous bacterial second messenger (Hengge, 2009) involved in the regulation of surface attachment, aggregation, and biofilm formation in a wide range of bacteria (Duvel et al., 2012). Moreover, c‐di‐GMP also interacts with the quorum sensing (QS) system (Sharma, Petchiappan, & Chatterji, 2014), a cell‐to‐cell communication phenomenon, involved in the regulation of genes that control biofilm formation, sporulation, bioluminescence, and virulence (Hammer & Bassler, 2003), and is mediated by a variety of small diffusible auto‐induced signaling molecules (Ueda & Wood, 2009). Taken together, these results suggest that the biofilm‐promoting effects of glucose on *P. aeruginosa* may be due to the Psl‐dependent upregulation of c‐di‐GMP.

The metabolomics analysis revealed that glucose treatment significantly altered metabolite production in *P. aeruginosa*. We found that glucose treatment mainly enhanced the production of 3‐hydroxypropionic acid and glucose‐6‐phosphate and reduced the production of myo‐inositol and glutamine (Figure 4). 3‐Hydroxypropionate is an intermediate of the 3‐hydroxypropionate and 3‐hydroxypropionate/4‐hydroxybutyrate cycle, two of the six pathways for autotrophic carbon dioxide fixation in microorganisms (Kumar, Ashok, & Park, 2013). However, few of the pathways described in the previous sections have been studied with glucose as a substrate. Therefore, the addition of exogenous glucose, which is a major carbon source, may increase the production of 3‐hydroxypropionate. The catalysis of glucose to glucose‐6‐phosphate by hexokinase is the first step of glycolysis (https://www.kegg.jp/kegg-bin). To some degree, the production of glucose‐6‐phosphate is positively corrected with the amount of the glucose substrate. As a consequence, the production of glucose‐6‐phosphate is promoted by the increase in exogenous glucose; Myo‐inositol is synthesized from glucose‐6‐phosphate using two sequentially acting enzymes: Inositol‐3‐phosphate synthase converts glucose‐6‐phosphate to inositol‐3‐phosphate, and then inositol monophosphatase dephosphorylates inositol‐3‐phosphate to generate myo‐inositol (Reynolds, 2009). In the inner biofilm, the cells prefer to acquire nutrition by transferring carbohydrate metabolism to glycolysis due to the lack of oxygen. Therefore, with the decrease in myo‐inositol, more glucose‐6‐phosphate will accumulate to participate in glycolysis to produce ATP. Similarly, the production of glutamine and methoxamedrine is probably also inhibited by *P. aeruginosa* in the same way. These findings suggest that targeting the enzymes associated with these metabolites may influence glucose‐induced biofilm growth and warrants further investigation.

**Figure A1:**
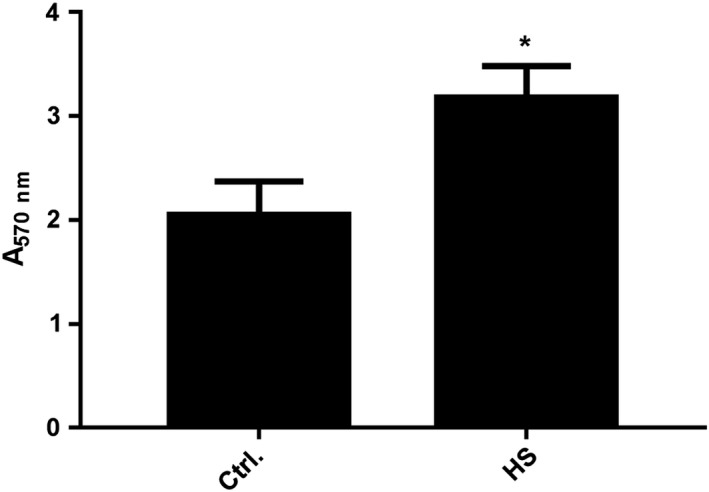
Effects of 2.5% human serum on PAO1 biofilm formation. *p < .05

Once the sugars (including polysaccharide and monosaccharide) are digested by gastrointestinal tract, the commonest existent form is glucose. And when patients are infected with bacteria, the circumstances around bacteria are blood or other body fluids also containing glucose. Moreover, published reports suggest that *P. aeruginosa* forms biofilms in the CF lung (Mah & O'Toole, 2001). And the CF patients are commonly complicated by glucose tolerance, hyperglycemia, or diabetes mellitus. The prevalence of CF‐related diabetes affects more than 1/4 CF patients over the age of 20 (Moran, 2000). It has recently been reported that uncontrolled hyperglycemia in diabetics increase airway glucose, providing a richer growth medium that facilitates *P. aeruginosa* infection (Baker et al., 2006). And the airway glucose is an important determinant of increased bacterial loads during diabetes (Gill et al., 2016). Because HoS is easy to be obtained and has similar effects on *P. aeruginosa* biofilms as human serum (Figure A1), many studies have tested the antimicrobial effects of agents by adding HoS to neutralize the differences between the in vitro properties of antibiotics. To simulate the body fluid environment of the human body, we detected the biofilm‐promoting activity of glucose in the presence of HoS. In this study, we found that the free glucose in the heat‐inactivated serum could be synergy with serum components enhancing *P. aeruginosa* biofilm formation and antibiotic resistance (Figures 5e and 6). These findings are in agreement with those of Samaranayake, Anil, Hashem, Vellappally, and Cheung (2015), who reported that human serum significantly upregulates biofilm‐related gene expression in *Candida* and enhances antifungal drug resistance on central venous catheters. Our results indicate that the effect of serum on *P. aeruginosa* biofilm formation may differ depending on the source.

## 5 CONCLUSION

This work adds to a growing understanding of the positive role of glucose in promoting *P. aeruginosa* biofilm formation. This is the first study to demonstrate that glucose causes metabolic changes associated with biofilm formation. Thus, our work sheds some light on the underlying mechanisms by which glucose enhances biofilm formation and identifies novel targets for developing strategies to counteract biofilm formation. Our findings also imply that higher dose of OFLX is needed to eliminate biofilms formed in the presence of glucose.

## CONFLICT OF INTERESTS

None declared.

## AUTHOR CONTRIBUTIONS

S.P. conceived, designed, and preformed experiments and contributed to the writing of the manuscript; W.Yanle. performed experiments, software and data analysis; L.Y. performed experiments and contributed to the writing of the manuscript. T.F performed experiments and software. C.L. and L.Z. performed experiments and edited final version of manuscript. W.Yong offered fund and conceived and administrated experiments and contributed to the writing and editing of the manuscript.

## ETHICAL APPROVAL

None required.

## Data Availability

All primer sequence data and the metabonomic analysis data are provided in Tables A1 and A2, respectively.

## Appendix 1

**Table A1:** Oligonucleotides used in this study

Name	Sequence (5’–3’)	Function
PslA‐MF1	ggaatctagaccttgagtcgGCTGGTTCTGGTAGAGCCGTC	pLP12 fusion
PslA‐MR1	CGGAACAGGATGTAGAGGTCGAACAGCGACAGGCTATCTACCGACT	Upstream of *pslA*
PslA‐MF2	agtcggtagatagcctgTCGCTGTTCGACCTCTACATCCTGTTCCG	pLP12 fusion
PslA‐MR2	ACAGCTAGCGACGATATGTCTCGATATAGCCGAAGCCGGT	Downstream of *pslA*
PslA‐TF	TCTCCAAGTGGGACGCCTACG	Deletion verification
PslA‐TR	AGTTCCTGCAACACGGTGGC	Deletion verification
pLP‐UF	GACACAGTTGTAACTGGTCCA	Insertion verification
pLP‐UR	CAGGAACACTTAACGGCTGAC	Insertion verification
pslA‐F	CGCTCACGGTGATTATGTTC	qPCR for *pslA*
pslA‐R	TACATGAACAACAGCAGGCA	qPCR for *pslA*
pelA‐F	ACAGCCAGGTAATGGACCTC	qPCR for *pelA*
pelA‐R	AAGCTGTCCAGGGTATCGAG	qPCR for *pelA*
alg44‐F	CTACCTTCTCGGCCAACCT	qPCR for *alg44*
alg44‐R	GTCAGGGTCCCTTTCATCTG	qPCR for *alg44*
16S rRNA‐F	GTGGTTCAGCAAGTTGGA	Housekeeping gene
16S rRNA‐R	CCTCAGTGTCAGTATCAGTC	Housekeeping gene

**Table A2:** Differentially expressed metabolites

ID	Peak	Similarity	rt	Count	Mass	Mean B^a^	Mean A^a^	VIP	*p*‐Value	*Q*‐value	Fold change	LOG_Foldchange
258	Glycine 2	900	10.9412,0	22	174	0.000636555	0.000905682	1.681654595	.021360277	.470350794	0.702845776	−0.508719938
530	Myristic acid	864	17.4935,0	22	117	0.000471343	0.000733843	2.026548698	.039435112	.495496165	0.642293626	−0.638695115
580	Methyl heptadecanoate	838	19.2904,0	22	74	0.00061175	0.001137859	2.602664342	.007154043	.392233078	0.537632544	−0.895307626
230	Ethanolamine	773	10.3913,0	22	174	0.000817073	0.002089325	1.882824161	.008936497	.412793867	0.391070269	−1.354500236
596	Myo‐inositol	725	19.7907,0	22	217	5.2432E−05	0.000127505	2.213988037	.032643332	.488588572	0.411215558	−1.282033246
537	Fructose 2	677	17.6171,0	16	73	0.001503234	0.000264768	2.298420367	.000201223	.062032217	5.677560028	2.505271054
351	Beta‐Alanine 2	641	12.565,0	19	248	2.14344E−05	4.88161E−05	1.005140441	.044227856	.499171803	0.439084406	−1.187429797
571	Gluconic acid 1	634	18.8501,0	22	73	0.001331955	0.000486406	2.181312099	.018254163	.46245845	2.738360012	1.45331213
126	3‐Hydroxypropionic acid 1	628	8.64861,0	15	147	0.000227447	3.86729E−05	1.89809182	.003786769	.320961262	5.881290599	2.556132777
564	Ascorbate	617	18.4939,0	22	117	0.000141637	0.000236679	1.729992969	.029429218	.484560963	0.598435492	−0.740732353
555	Sedoheptulose	575	18.229,0	22	204	0.000143338	8.29165E−05	1.443433138	.019096923	.46482548	1.728704982	0.789691682
263	Unknown	553	11.028,0	22	174	0.000279223	0.000424518	1.915006827	.011902268	.435631776	0.657739563	−0.604411643
88	Unknown	473	7.87172,0	15	154	5.92006E−05	2.74291E−05	2.172911772	.019095927	.464822792	2.158313081	1.109904155
774	Dioctyl phthalate	464	23.6597,0	22	149	0.000116688	0.000161225	1.681681562	.023504702	.474694377	0.723760618	−0.466415487
306	2,3‐Dimethylsuccinic acid	413	11.7677,0	22	221	0.000942685	0.000548862	1.104732534	.028256141	.482882576	1.717528774	0.780334269
341	*N*‐Ethylglycine 1	407	12.322,0	20	142	0.000365908	0.001677752	2.011080769	.00030861	.072366056	0.218094053	−2.196977662
815	Androstanediol	394	24.7242,0	22	215	0.000645328	0.0009989	2.361081193	.013021745	.44196591	0.646039227	−0.630306329
487	Unknown	385	16.1412,0	4	73	9.78762E−05	1.33238E−09	1.927482839	.046504973	.501498802	73,459.62957	16.164664
390	4‐Acetamidobutyric acid 2	381	13.516,0	21	174	0.000197373	0.000376937	1.067381356	.021187565	.469966567	0.523624016	−0.933396828
84	Analyte 101	338	7.82067,0	15	147	6.42091E−05	1.96573E−05	2.191516203	.025125818	.477710913	3.266430242	1.70771483
652	Unknown	338	21.1104,0	11	183	0.000352283	1.33238E−09	2.158139288	.041964778	.497534124	264,401.0212	18.01236822
459	Unknown	332	15.2644,0	4	73	1.28223E−09	0.000450593	1.798650896	.036228371	.492533753	2.84565E−06	−18.42280931
378	Unknown	328	13.2206,0	22	174	0.00022994	0.0003076	1.986934543	.005907729	.372603568	0.747529218	−0.419798125
192	Unknown	326	9.71867,0	10	103	6.4026E−05	6.36615E−06	1.750377931	.028741275	.483591877	10.05726382	3.330165953
757	Salicin	322	23.2598,0	22	268	0.001969318	0.002926527	2.097665134	.037881974	.494119578	0.67291969	−0.571493758
755	Neohesperidin	316	23.2301,0	22	259	0.002558287	0.003988477	2.278077859	.015242434	.45214943	0.641419517	−0.640659842
651	Unknown	311	21.0898,0	11	127	0.000291231	1.33238E−09	2.273471926	.021554222	.470775646	218,579.3535	17.73779761
814	dihydrotestosterone	307	24.7072,0	22	254	0.000682601	0.001060085	2.368585174	.015266066	.45224402	0.64391169	−0.635065252
157	Unknown	304	9.12182,0	11	267	0.000379069	4.13821E−05	2.697230347	9.50082E−06	.006683556	9.160218651	3.195382035
690	Unknown	302	21.7667,0	22	74	0.001128332	0.001688802	2.30097913	.009361508	.416774798	0.668126012	−0.581807866
752	Analyte 867	300	23.1975,0	22	113	0.002689158	0.004064865	2.125973172	.042392132	.497855921	0.661561578	−0.596052646
454	Unknown	299	15.2144,0	4	155	1.28223E−09	3.31542E−05	1.796567609	.036471165	.492774945	3.86747E−05	−14.65825047
751	Analyte 866	290	23.1883,0	22	226	0.014606521	0.021855725	2.109873509	.044279473	.499207322	0.668315535	−0.581398685
165	Unknown	289	9.20858,0	20	174	0.000165965	0.000280953	1.587651675	.026109306	.479456879	0.590723043	−0.759446204
760	Analyte 875	287	23.3164,0	22	173	0.003045102	0.004570994	2.342115269	.014707448	.449938128	0.666179422	−0.586017305
273	4‐Acetylbutyric acid 2	285	11.1765,0	21	174	4.44156E−05	9.38684E−05	1.309081961	.005491246	.364658429	0.473168653	−1.079573597
711	Unknown	285	22.4259,0	20	226	0.000201512	0.000429821	2.096817887	.02126821	.470146675	0.468826647	−1.092873522
770	Analyte 885	276	23.5711,0	16	145	9.06817E−05	0.00019568	1.310305407	.042568261	.497986784	0.463418625	−1.109612068
483	Glutamine 2	269	16.024,0	12	154	7.28123E−05	0.001285245	2.016098221	.028042727	.482563457	0.056652467	−4.141717403
667	Unknown	258	21.3307,0	21	98	0.000197059	0.000289329	1.70059336	.008320611	.406463982	0.681091293	−0.554079906
694	Glucose−6‐phosphate 2	258	21.8499,0	12	86	0.000205605	3.82541E−05	1.684651431	.04010425	.496058575	5.374703563	2.426185186
87	Analyte 104	252	7.87963,0	18	89	3.6662E−05	9.50563E−05	1.643073189	.03634495	.492649936	0.385687373	−1.374496179
168	Unknown	247	9.26721,0	17	281	6.1893E−05	2.01719E−05	1.45803412	.005665154	.368076045	3.068280644	1.617430447
607	Analyte 701	240	20.0942,0	6	87	4.73497E−05	0.000577686	1.693128043	.042242217	.497743729	0.081964356	−3.608859529
281	Allylmalonic acid	234	11.3282,0	20	341	0.000138678	6.8898E−05	1.388553557	.012405111	.438595571	2.012805865	1.009208031
479	Methoxamedrine 2	224	15.9526,0	6	166	5.29336E−05	0.00033931	1.965510146	.035962702	.492266383	0.156003567	−2.680349077
709	Analyte 819	208	22.3485,0	5	152	1.28223E−09	3.20059E−05	2.175696958	.019925125	.466977373	4.00623E−05	−14.60739434
282	Analyte 319	206	11.357,0	22	227	0.000111597	0.00020074	1.481083252	.049254692	.504321124	0.555931028	−0.847022189
524	Analyte 607	200	17.2762,0	22	74	0.000424919	0.000888424	2.139910283	.012643434	.439930975	0.478284016	−1.064060517
754	Biotin	196	23.2218,0	22	201	0.000838393	0.001251854	2.182683791	.030664975	.486201342	0.669720659	−0.578368623
753	Analyte 868	161	23.2078,0	18	212	0.000712289	0.001480437	1.222977586	.012945675	.4415648	0.481134531	−1.05548775
7	Analyte 9	0	6.36885,0	8	159	0.000253871	1.33238E−09	1.842253232	.03915136	.495252265	190,539.333	17.53972932
8	Analyte 10	0	6.39045,0	22	102	0.014832721	0.008723585	2.520788492	.012259819	.437760121	1.700301099	0.76579025
47	Analyte 55	0	7.018,0	15	117	6.82734E−05	0.000187564	2.004841093	.003449839	.309299434	0.363999623	−1.457991137
769	Analyte 884	0	23.5743,0	10	110	0.000132283	1.33238E−09	2.762715307	.000591452	.10401739	99,282.83028	16.59925662

a A and B represent the *P. aeruginosa* in the absence and presence of 4% glucose, respectively.
